# Reveal the correlation between hub hypoxia/immune-related genes and immunity and diagnosis, and the effect of SAP30 on cell apoptosis, ROS and MDA production in cerebral ischemic stroke

**DOI:** 10.18632/aging.205339

**Published:** 2023-12-27

**Authors:** Yue Cao, Wanmei Yao, Rongrong Lu, Huan Zhao, Wenyi Wei, Xiaolei Lei, Zheng Zhang, Biwang Liu

**Affiliations:** 1 College of Basic Medical Sciences, Shanxi University of Chinese Medicine, Jinzhong, Shanxi 030619, China; 2 Department of Pharmacy, The Hospital of Shanxi University of Chinese Medicine, Taiyuan, Shanxi 140100, China; 3 School of Fushan, Shanxi University of Chinese Medicine, Jinzhong, Shanxi 030619, China

**Keywords:** cerebral ischemic stroke, immune, hypoxia, SAP30

## Abstract

Background: Cerebral ischemic stroke (CIS) is a common cerebrovascular disease. The purpose of this study was to investigate the potential mechanism of hypoxia and immune-related genes in CIS.

Methods: All data were downloaded from public databases. Hub mRNAs was identified by differential expression analysis, WGCNA analysis and machine learning. Hub mRNAs were used to construct the classification models. Pearson correlation analysis was used to analyze the correlation between hub mRNAs and immune cell infiltration. Finally, the SAP30 was selected for verification in HMC3 cells.

Results: The SVM, RF and DT classification models constructed based on 6 hub mRNAs had higher area under the curve values, which implied that these classification models had high diagnostic accuracy. Pearson correlation analysis found that Macrophage has the highest negative correlation with CCR7, while Neutrophil has the highest positive correlation with SLC2A3. Drug prediction found that ruxolitinib, methotrexate, resveratrol and resatorvid may play a role in disease treatment by targeting different hub mRNAs. Notably, inhibition of SAP30 expression can reduce the apoptosis of HMC3 cells and inhibit the production of ROS and MDA.

Conclusion: The identification of hub miRNAs and the construction of classification diagnosis models provide a theoretical basis for the diagnosis and management of CIS.

## INTRODUCTION

Cerebral ischemic stroke (CIS) is a common cerebrovascular disease in the department of neurology. It is a series of physiological and pathological changes caused by the interruption of cerebral blood circulation, which leads to the death of brain tissues and cells due to ischemia and hypoxia [1]. It also has the characteristics of high incidence, high disability rate and high mortality [2]. At present, the clinical diagnosis and treatment of CIS is still very complex, and most patients cannot be treated promptly, quickly and effectively. Therefore, continuous understanding of the potential molecular mechanism of CIS and exploration of novel biomarkers are conducive to the diagnosis and management of CIS.

The immune system plays an important role in the pathophysiology of stroke [3]. After CIS, many immune cell disorders, including Macrophages, Neutrophils, etc., are involved in the development of the disease with extremely complex effects [4]. The immune system is also involved in regulating brain repair after CIS [5]. A study has demonstrated the role of astrocytic IL-6-mediated negative immune regulation in promoting neurovascular regeneration and functional recovery after stroke [6]. Insufficient oxygen supply is an important cause of CIS [7]. The hypoxic induction factor-1α (HIF-1α) is considered to be a key regulator of oxygen homeostasis. It may regulate the inflammatory response through NLRP3 inflammatory small body composite, which affects the death of apoptosis and thermal cells after stroke [8]. Therefore, exploring the genes related to immunity and hypoxia in CIS is helpful to understand the molecular mechanism of the disease and contribute to the management of the disease.

Weighted gene co-expression network analysis (WGCNA) is a systems biology approach used to describe correlation patterns among genes in microarray samples [9]. It can be used to find clusters (modules) of genes that are highly associated with disease and identify candidate molecular biomarkers. Machine learning in biomedicine has a profound impact on disease detection, diagnosis and treatment [10]. In recent years machine learning algorithms have also been used to analyze the results of biomedical datasets, including support vector machines (SVM) random forests (RF) and decision trees (DT) [11, 12]. So far, few studies have combined WGCNA and machine learning algorithms to identify hypoxia and immune markers related to CIS. Therefore, the purpose of this study is to identify the important hypoxia and immune hub mRNAs related to CIS and construct diagnostic classification models through differential expression analysis, WGCNA and machine learning algorithm. In order to further understand the molecular mechanism of hub mRNAs, we also constructed transcription factors (TFs) and competing endogenous RNA (ceRNA) regulatory networks. In addition, drugs related to hub mRNAs were screened and molecular docking was carried out, hoping to provide a new perspective for the diagnosis, treatment and research of CIS.

## MATERIALS AND METHODS

### CIS data screening and preprocessing

Keywords “Ischemic stroke” and “Homosapiens” were used to screen gene expression data in Gene Expression Omnibus (GEO) database [13]. Studies at the cell line or animal level, single-sample studies, and duplicate or overlapping studies were then excluded. The datasets with no less than 5 samples and containing normal control population were included in this study. Finally, GSE58294 (blood samples from 69 CIS patients and 23 normal controls) and GSE16561 (blood samples from 39 CIS patients and 24 normal controls) datasets were selected for analysis. GPL platform annotation file was used to annotate gene expression profiles and convert gene probes into gene symbols. Multiple probes corresponding to the same gene were averaged. GSE58294 dataset was used as the experimental group, and GSE16561 dataset was used as the verification group. In addition, we downloaded the miRNA dataset GSE95204 (blood samples from 3 CIS patients and 3 normal controls) related to acute ischemic stroke. Detailed information of the selected datasets in this study is shown in Supplementary Table 1.

### Collection of immune and hypoxia-related genes

Immune-related genes (IRGs) were retrieved from the immunology database IMMPORT (https://immport.niaid.nih.gov), and a comprehensive list of IRGs was downloaded. A total of 1,793 IRGs were obtained. Hypoxia-related genes (HRGs) were extracted from the marker gene set in MSigDB (https://www.gseamsigdb.org/gsea/msigdb/) database. A total of 200 HRGs were obtained.

### Identification of differentially expressed mRNAs (DEmRNAs)

Differential expression analysis of mRNA in the GSE58294 and GSE16561 datasets was performed using the limma package. False discovery rate (FDR) <0.05 and |log2fold change| (|log2FC|) >0.2 were used as the cut-off criterion for identifying DEmRNAs. Subsequently, the “ggplot” package was used to draw the volcanic maps.

### WGCNA

The WGCNA package was used to analyze the mRNA in the top 25% of the coefficient of variation in GSE58294 dataset, and a scale-free gene co-expression network was constructed. The “hclust” function and the “pickSoftThreshold” function were used to detect outliers and select the appropriate soft threshold power (β) regulator, respectively. Subsequently, the adjacency matrix is calculated according to the kernel value [14]. Genes with similar expression patterns were grouped together, and modules were divided according to the “cutreeDynamic” function with default parameters. Then, the dynamic tree cutting method was used to merge the modules with the similarity <25%. To determine the most related module with CIS, the “Moduleeigengenes” function was used to calculate the module eigengene (ME) of each module. Subsequently, Pearson correlation method was used to analyze the correlation between ME and CIS. The module with the highest correlation with CIS was selected as the hub module. Finally, candidate hub mRNAs were selected according to the module connectivity (MM) >0.2 and clinical trait relationship (GS) >0.5 of each gene in hub modules [15].

### Identification and functional enrichment analysis of intersection mRNAs

The intersection of the DEmRNAs in the GSE58294 dataset, the DEmRNAs in the GSE16561 dataset, the candidate hub mRNAs in the WGCNA and the set of IRGs and HRGs were taken. In order to understand the biological functions of intersection mRNA, GO and KEGG functional enrichment analysis was performed based on DAVID database (https://david.ncifcrf.gov/). GO functional analysis includes 3 categories, namely biological process (BP), cellular component (CC) and molecular function (MF). *P* < 0.05 was considered statistically significant. In addition, a protein-protein interaction (PPI) network was constructed based on STRING database (https://cn.string-db.org/) to study the regulatory relationship between intersection mRNAs.

### Identification of hub mRNAs and construction of classification models based on machine learning

Intersection mRNAs were analyzed using LASSO regression in the glmnet package to screen out important mRNAs. In LASSO analysis, family parameter was set to binomial, alpha parameter was set to 1, nfolds parameter was set to 10, type parameter was set to class, s parameter was set to lambdo.min, and others were the default parameters. Then, the random forest algorithm in the randomForest package was used to rank the importance of the mRNAs screened by LASSO from largest to smallest according to the mean decreased accuracy value. One mRNA was added at a time from top to bottom according to the ranking order. Subsequently, random forest algorithm and 10-fold cross validation were used to obtain the optimal mRNA quantity.

SVM, RF and DT classification models were constructed based on the selected hub mRNAs using e1071, randomForest and rpart packages, respectively. The probability parameter of the e1071 package used to construct the SVM model was set to TRUE, while others were the default parameters. When the randomForest package constructs the RF model, the ntree parameter was set to 500, the importance parameter was set to TRUE, the proximity parameter was set to TRUE, and others were the default parameters. The method parameter of the rpart package used to construct the DT model was set to class, while others were the default parameters. In addition, multilayer perceptron (MLP) classification model was also constructed based on the selected hub mRNAs using caret package. The method parameter of the caret package used to construct the MLP model was set to mlp, while others were the default parameters. The area under the curve (AUC) values of the receiver operating characteristic (ROC) of the classification models were used to evaluate the potential diagnostic ability of classification models. In the ROC curve, the greater AUC, the higher the diagnosis accuracy [16]. AUC > 0.7 indicates good diagnostic accuracy.

### Analysis of immune cell infiltration in GSE58294 dataset

Gene sets marking each immune cell type were obtained from Charoentong’s study [17]. The ssGSEA algorithms were used to quantify the level of immune cell infiltration in the immune microenvironment. In addition, the CIBERSORT method also was used to evaluate the proportion of various immune cell types in the sample. The Wilcoxon test was used to statistically analyze the difference of immune cell infiltration level between CIS group and normal control group. In order to further understand the correlation between hub mRNAs and immune cell infiltration, we also performed Pearson correlation analysis.

### Construction of TFs regulatory network and ceRNA regulatory network

The TFs related to hub mRNAs were queried based on TRRUST database (https://www.grnpedia.org/trrust/) to construct TFs regulatory network. In addition, to further understand the molecular mechanism of hub mRNA, a ceRNA regulatory network was constructed. The target miRNAs of hub mRNAs were predicted based on ENCORI (http://starbase.sysu.edu.cn/index.php) and miRDB (http://mirdb.org/) databases. Meanwhile, differential expression of miRNA in GSE95204 dataset was analyzed. The screening threshold was *P* < 0.05. Then, the intersection of differentially expressed miRNAs (DEmiRNAs) negatively regulated with hub mRNAs and targeted miRNAs were screened out. The lncRNAs associated with intersection miRNAs were searched based on ENCORI database, and then the correlation between these lncRNAs and hub mRNAs was predicted. Subsequently, lncRNAs associated with hub mRNAs were screened out (*P* < 0.05). Finally, the ceRNA regulatory network was constructed based on the selected miRNAs, lncRNAs and hub mRNAs.

### Drug prediction

Drugs related to hub mRNAs were screened based on DGId database (https://dgidb.org/), hoping to provide a new perspective for the treatment and research of CIS. In addition, we also carried out molecular docking between some drugs and hub mRNAs. The purpose of molecular docking is to find the best binding site between the compound and the target gene when the binding energy is the lowest. Binding energy less than −1.19423 kcal/mol (Note: −1.19423 kcal/mol = −5.0 kJ/mol) is the basis for screening candidate targets of active ingredients [18, 19]. The 3D structures of target proteins and drugs were downloaded from RCSB PDB (http://www.rcsb.org/pdb/home/home.do) and pubchem (https://pubchem.ncbi.nlm.nih.gov/) databases, respectively. Protein receptors were treated in PyMol to remove water molecules, and then hydrogenation and other pretreatments in AutoDockTools. Drug components are also pretreatments in AutoDockTools. Subsequently, the molecular docking calculation was performed, and the docking results were visualized using PyMol software.

### Cell experiment

HMC3 cells were selected for *in vitro* validation. HMC3 was treated with oxygen-glucose deprivation reperfusion (OGD/R) to induce ischemia/reperfusion (I/R) injury models (4 h oxygen-glucose deprivation and 24 h reoxygenation normal culture) *in vitro* to simulate ischemic stroke. Real time-PCR was used to detect the relative expression levels of CCR7, S100A12, SAP30 and SLC2A3 in the HMC3-OGD/R model group and the HMC3 control group. The primers used for real time-PCR are shown in Supplementary Table 2. The relative expression levels of CCR7, S100A12, SAP30 and SLC2A3 were calculated using the 2^−ΔΔCt^ method [20]. SAP30 was selected to knockdown in HMC3 cells. Then, the expression of SAP30 was detected by real time-PCR to screen out effective interference targets. Subsequently, construction of OGD/R model was continued to form HMC3-si-SAP30-OGD/R cells. The relative contents of reactive oxygen species (ROS) and malondialdehyde (MDA) in each group were detected by ROS and MDA kit of Nanjing Jiancheng. In addition, flow cytometry was used to detect the effect of SAP30 on cell apoptosis.

### Statistical analysis

All statistical analyses were processed by R software. The limma package was used to identify DEmRNAs based on the screening criteria FDR <0.05 and |log2FC| >0.2. The Wilcoxon test was used to statistically analyze the difference of immune cell infiltration level between CIS group and normal control group. The relative expression levels of CCR7, S100A12, SAP30 and SLC2A3 were calculated using the 2^−ΔΔCt^ method. *P* < 0.05 was considered significant difference.

### Data availability statement

All data generated or analyzed during this study are included in this published article.

## RESULTS

### Identification of DEmRNAs

**Figure 1:**
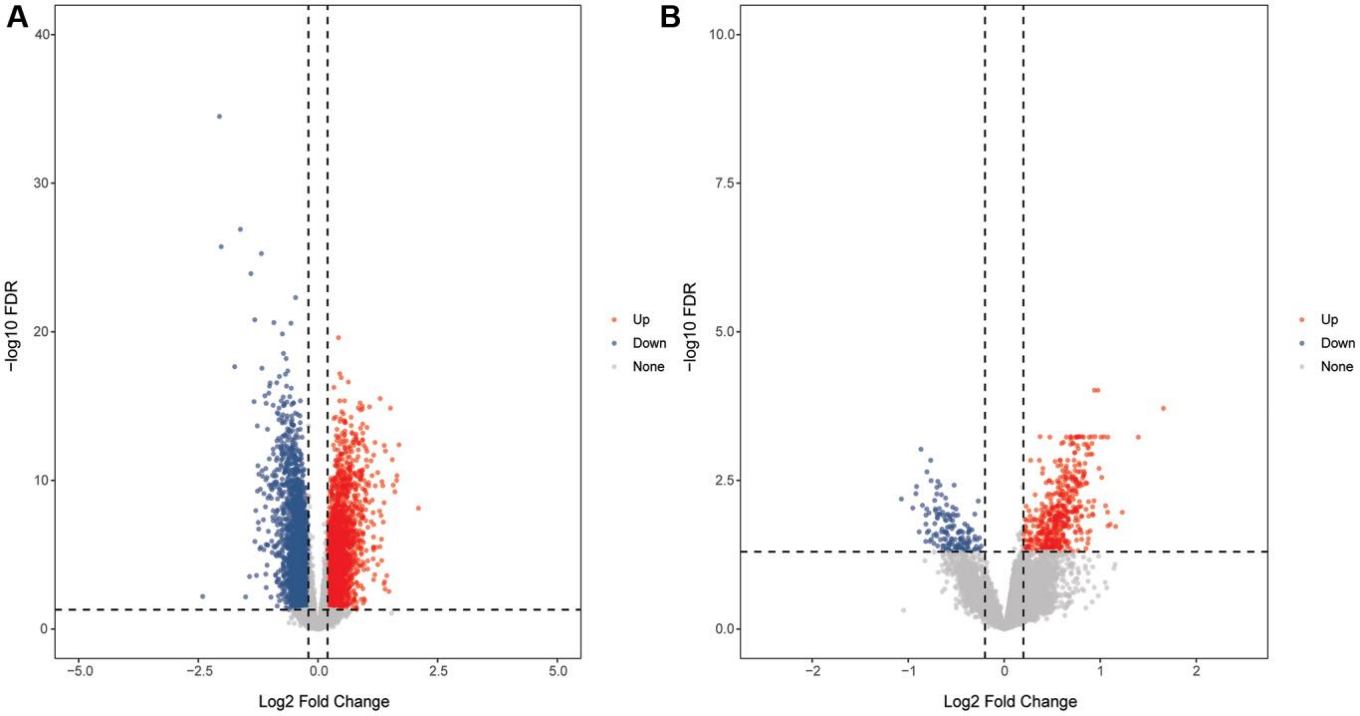
**Volcanic maps of DEmRNAs.** (**A**) Volcanic map of DEmRNAs in the GSE58294 dataset; (**B**) Volcanic map of DEmRNAs in the GSE16561 dataset.

DEmRNAs in GSE58294 and GSE16561 datasets were screened using the limma package according to the screening criteria FDR <0.05 and |log2FC| >0.2. In the GSE58294 dataset, 6,145 DEmRNAs were identified in the CIS group compared with the control group, of which 3,109 DEmRNAs were up-regulated and 3,036 DEmRNAs were down-regulated. In the GSE16561 dataset, 648 DEmRNAs were identified in the CIS group compared with the control group, of which 497 DEmRN were up-regulated and 151 DEmRNAs were down-regulated. The volcanic maps of DEmRNAs in the GSE58294 and GSE16561 datasets are shown in Figure 1A and 1B.

### Identification of candidate hub mRNAs based on WGCNA

**Figure 2:**
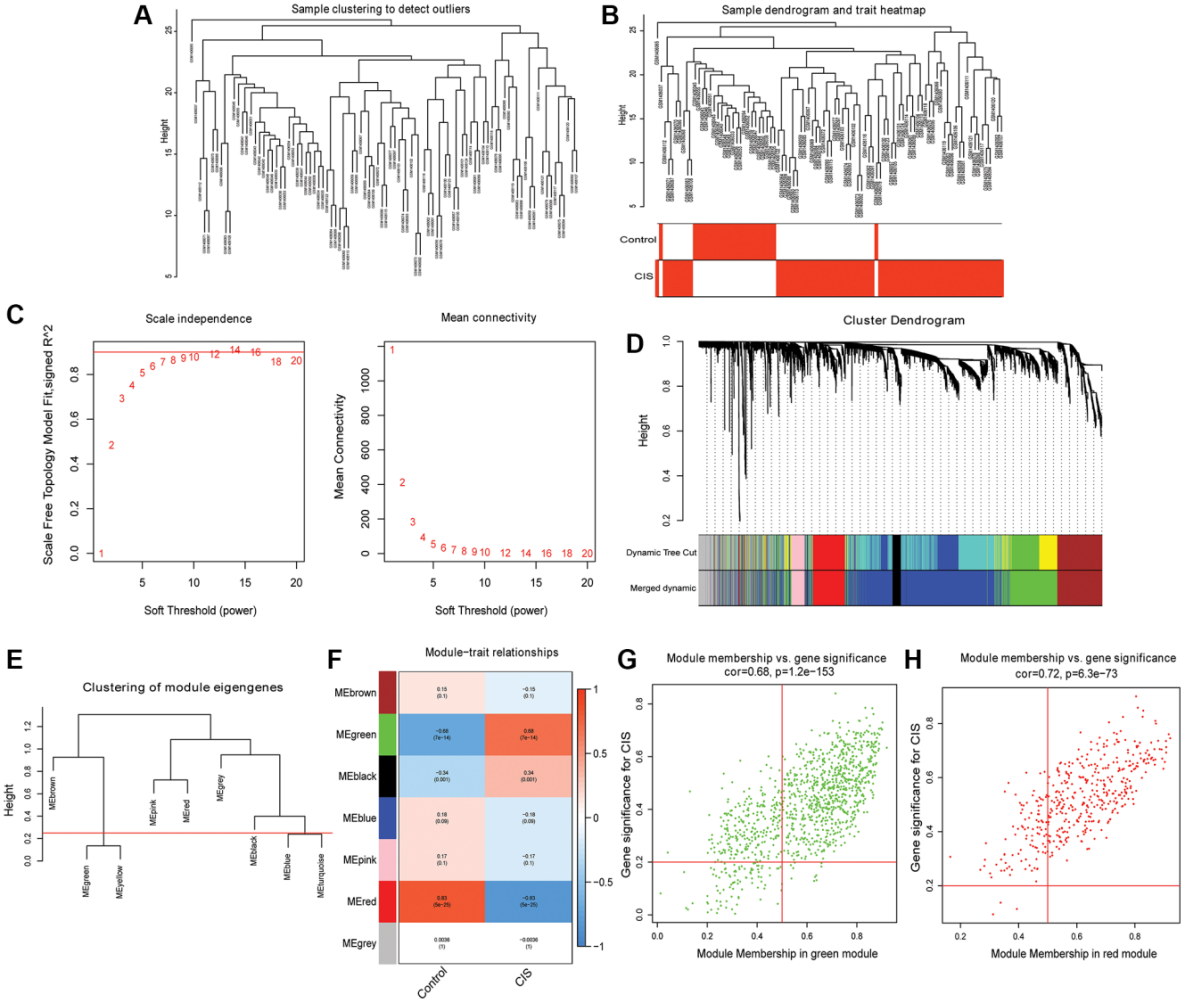
**Identification of hub modules and candidate hub mRNAs based on WGCNA.** (**A**) Sample clustering dendrogram to detect outliers; (**B**) Sample clustering dendrogram and trait heatmap; (**C**) Scale-free fitting index and average connectivity for different soft threshold power (β); (**D**) mRNA is divided into different modules by hierarchical clustering, and different colors represent different modules; (**E**) Modules with dissimilarity <25% are merged; (**F**) Heatmap of correlation between ME and CIS; (**G**) Scatter plot of mRNAs in green module; (**H**) Scatter plot of mRNAs in red module.

The “hclust” function was used to cluster the samples, and no outlier samples were found (Figure 2A and 2B). When β = 7, the topology is approximately scale-free (Figure 2C). After constructing the cluster dendrogram, the minimum number of mRNAs in the module was set to 100, and 8 modules were isolated. Then, the dynamic tree cutting method was used to merge the modules with the similarity <25%. Finally, 6 modules were determined (Figure 2D and 2E). Pearson correlation analysis showed that the red module had the highest negative correlation with CIS (Pearson r = −0.83), and the green module had the highest positive correlation with CIS (Pearson r = 0.68) (Figure 2F). Therefore, the green and red modules are considered hub modules. Based on GS >0.2 and MM >0.5, 350 mRNAs were obtained in the red module (Figure 2G), and 777 mRNAs were obtained in the green module (Figure 2H). A total of 1,127 mRNAs were considered as candidate hub mRNAs.

### Identification and functional enrichment analysis of intersection mRNAs

**Figure 3:**
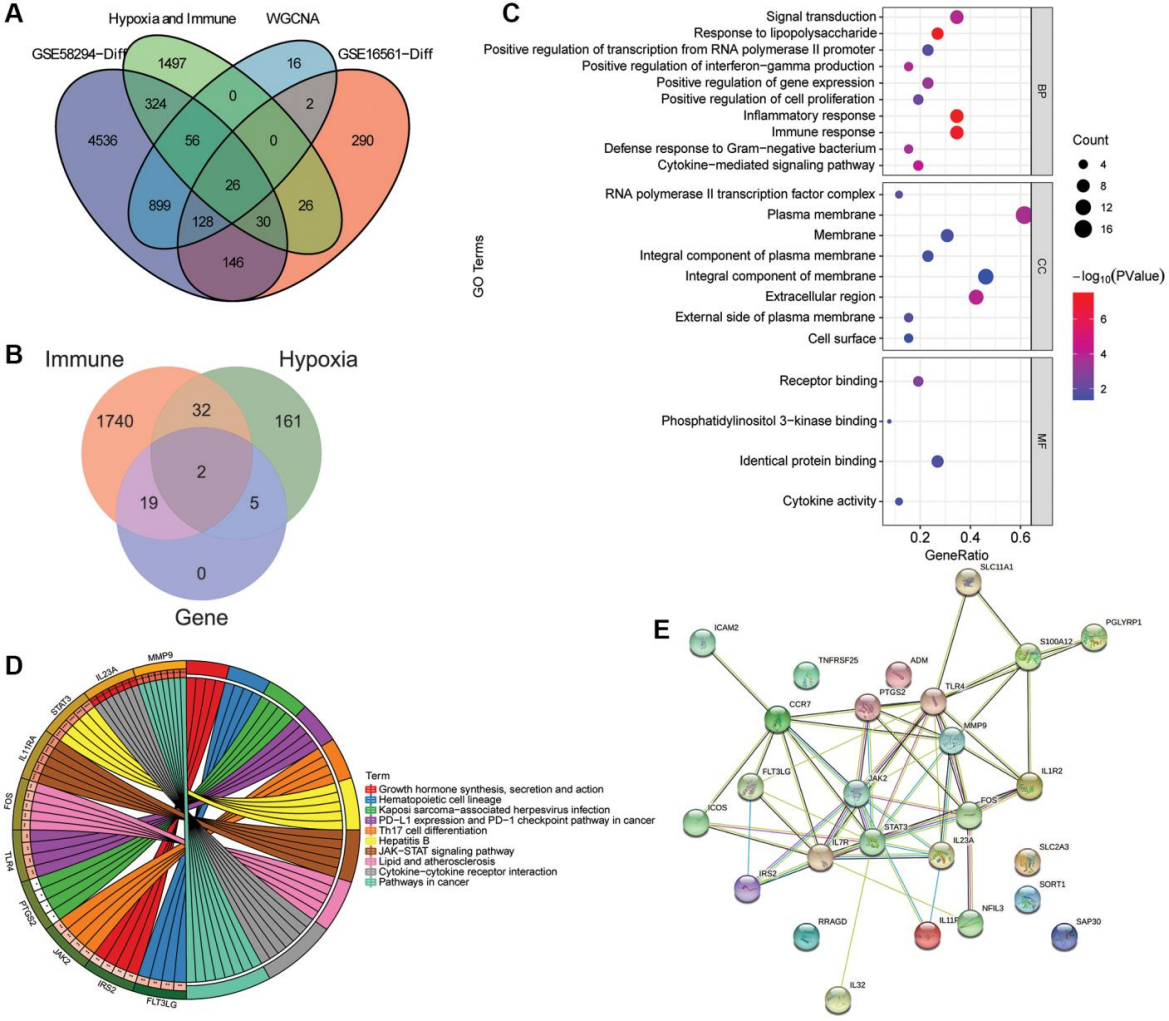
**Identification and functional enrichment analysis of intersection mRNAs.** (**A**) Venn diagram of intersection of the DEmRNAs in the GSE58294 dataset, the DEmRNAs in the GSE16561 dataset, the candidate key mRNAs in the WGCNA and the set of IRGs and HRGs; (**B**) Venn diagram of the intersection mRNAs, IRGs and HRGs; (**C**) GO functional enrichment analysis of intersection mRNAs; (**D**) KEGG functional enrichment analysis of intersection mRNAs; (**E**) A PPI network was constructed based on STRING database to study the regulatory relationship between intersection mRNAs.

A total of 26 intersection mRNAs were obtained (Figure 3A), of which 19 were IRGs, 5 were HRGs, and 2 were the intersection of IRGs and HRGs (Figure 3B). In GO-CC, the intersection mRNAs were mainly distributed in plasma membrane, integral component of membrane. In GO-MF, the intersection mRNAs were mainly involved in identical protein binding and receptor binding. In GO-BP, the intersection mRNAs were mainly involved in the regulation of inflammatory response and immune response (Figure 3C). KEGG analysis showed that intersection mRNAs were significantly enriched in cytokine-cytokine receptor interaction, hepatitis B and JAK-STAT signaling pathway (Figure 3D). STAT3 and TLR4 were found to have more interacting genes in PPI network (Figure 3E). In addition, 2 pairs with interaction scores >0.99 were JAK2 and STAT3 (interaction score = 0.999), FOS and STAT3 (interaction score = 0.998), respectively.

### Identification of hub mRNAs and construction of diagnostic classification models based on machine learning

**Figure 4:**
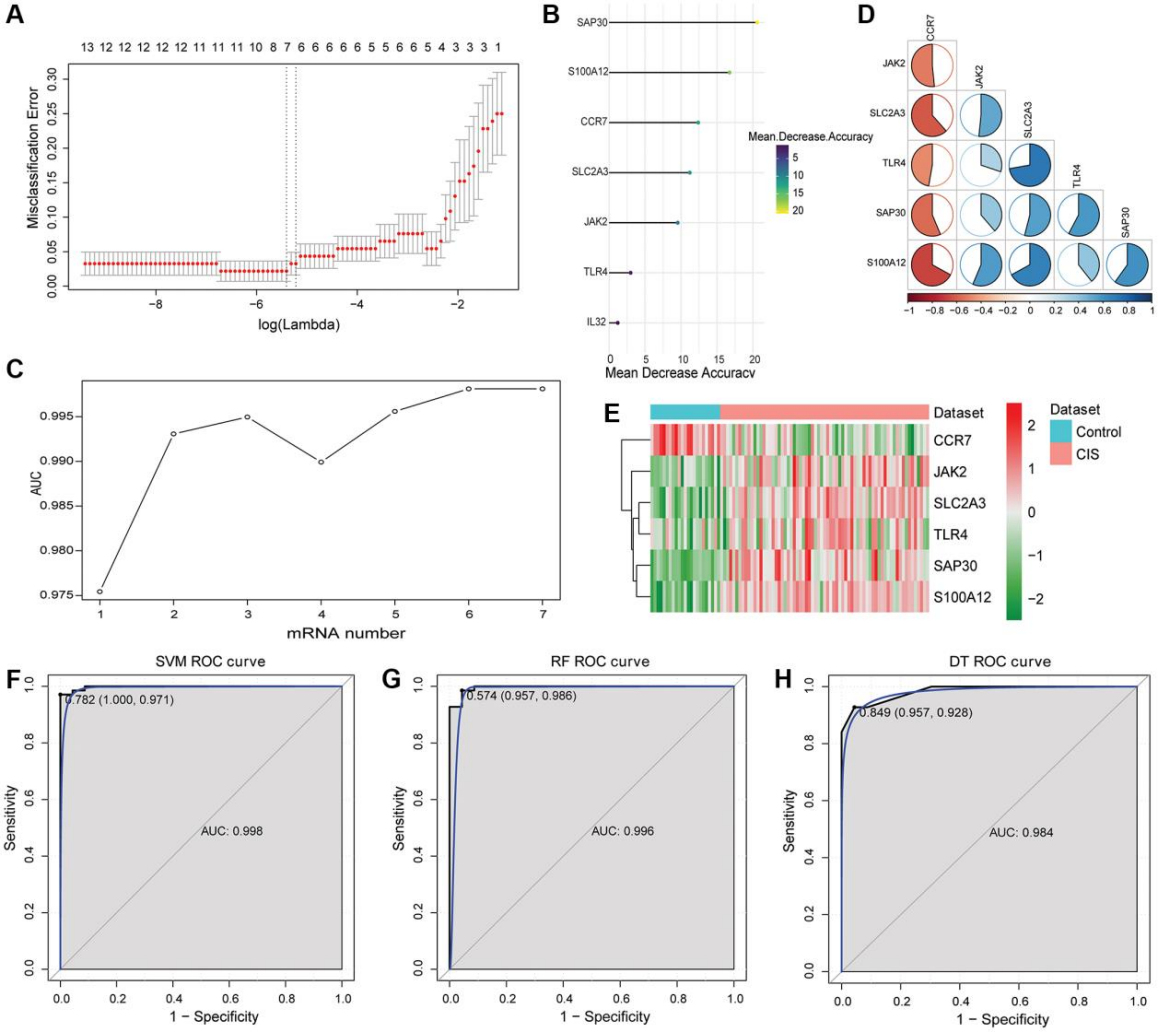
**Identification of hub mRNAs and construction of SVM, RF and DT classification models.** (**A**) LASSO regression analysis was performed on 26 intersection mRNAs; (**B**) Mean decreased accuracy sorting of SAP30, S100A12, CCR7, SLC2A3, JAK2, TLR4 and IL32; (**C**) Trend chart of AUC with the increase of DEmRNA quantity; (**D**) Correlation between SAP30, S100A12, CCR7, SLC2A3, JAK2 and TLR4. Red and blue represent positive and negative correlations, respectively. (**E**) Expression heatmap of SAP30, S100A12, CCR7, SLC2A3, JAK2 and TLR4; (**F**) ROC curve of SVM classification model in GSE58294 dataset; (**G**) ROC curve of RF classification model in GSE58294 dataset; (**H**) ROC curve of DT classification model in GSE58294 dataset.

After LASSO analysis, 7 mRNAs were screened out from 26 intersection mRNAs (Figure 4A). The 7 mRNAs were ranked according to mean decreased accuracy values (Figure 4B). One mRNA was added at a time from top to bottom according to the ranking order. Then, RF algorithm was used for classification, and AUC was obtained by 10-fold cross validation. The results showed that AUC reached the maximum value when the number of mRNAs reached 6 (Figure 4C). These 6 mRNAs (CCR7, JAK2, S100A12, SAP30, SLC2A3 and TLR4) were considered as hub mRNAs, among which S100A12, JAK2, CCR7 and TLR4 were IRGs, and SAP30 and SLC2A3 were HRGs. The correlation between CCR7, JAK2, S100A12, SAP30, SLC2A3 and TLR4 is shown in Figure 4D. Compared with the control, JAK2, S100A12, SAP30, SLC2A3 and TLR4 were up-regulated and CCR7 was down-regulated in the CIS (Figure 4E).

**Figure 5:**
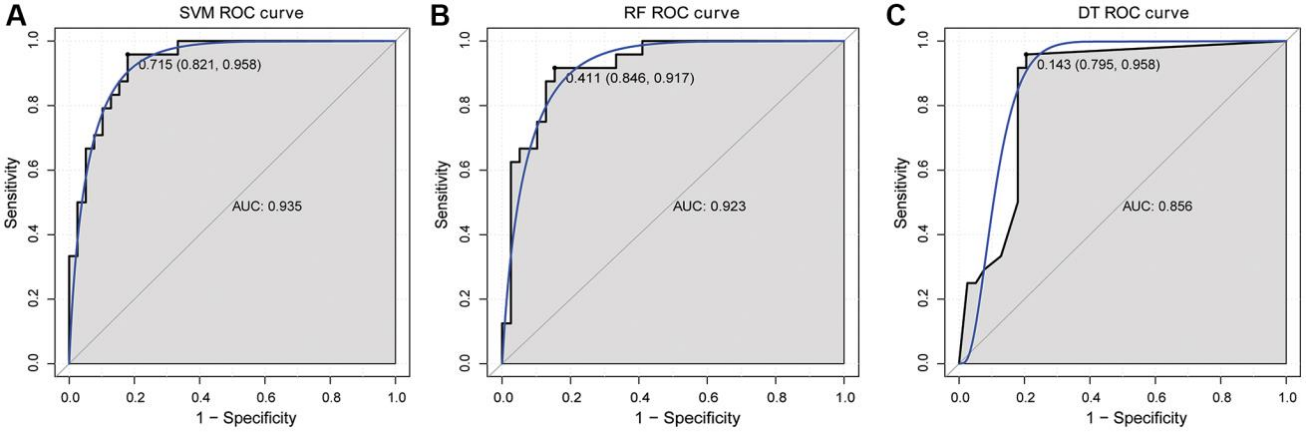
ROC curve validation of SVM (**A**), RF (**B**) and DT (**C**) classification models in GSE16561 dataset.

Subsequently, SVM, RF and DT classification models were constructed based on 6 hub mRNAs in GSE58294 dataset (Figure 4F–4H). The results showed that the AUC values of SVM, RF and DT classification models were 0.998, 0.996 and 0.984, respectively, which implied that these three classification models had high diagnostic accuracy. Subsequently, ROC analysis of the 6 hub mRNAs showed that the AUC >0.7 (Supplementary Figure 1), which implied that they also had high diagnostic accuracy. However, the AUC of 6 hub mRNAs was less than SVM, RF and DT classification models, which implied that the diagnostic accuracy of classification models were higher than that of single hub mRNAs. In addition, e1071, randomForest and rpart packages were also used to construct SVM, RF and DT classification models based on the 6 hub mRNAs in GSE16561 dataset with the same parameters to verify the diagnostic value. The results showed that the AUC values of SVM, RF and DT classification models were 0.935, 0.923 and 0.856, respectively (Figure 5). At the same time, ROC analysis of 6 hub mRNAs was also performed in the GSE16561 dataset (Supplementary Figure 2). The results showed that the AUC >0.7 of 6 hub mRNAs, but less than the AUC value of classification models. This again indicates that the classification models based on the 6 hub mRNAs has high diagnostic accuracy, which may be beneficial to the diagnosis and management of CIS. Moreover, the MLP classification model was constructed based on the 6 hub mRNAs in the GSE58294 (Supplementary Figure 3A) and GSE16561 (Supplementary Figure 3B) datasets. The results showed that the AUC values of the MLP classification model in the GSE58294 and GSE16561 datasets were 0.899 and 0.819 respectively, which implied that the MLP classification model based on the 6 hub mRNAs also had high diagnostic accuracy.

### Analysis of immune cell infiltration in CIS

**Figure 6:**
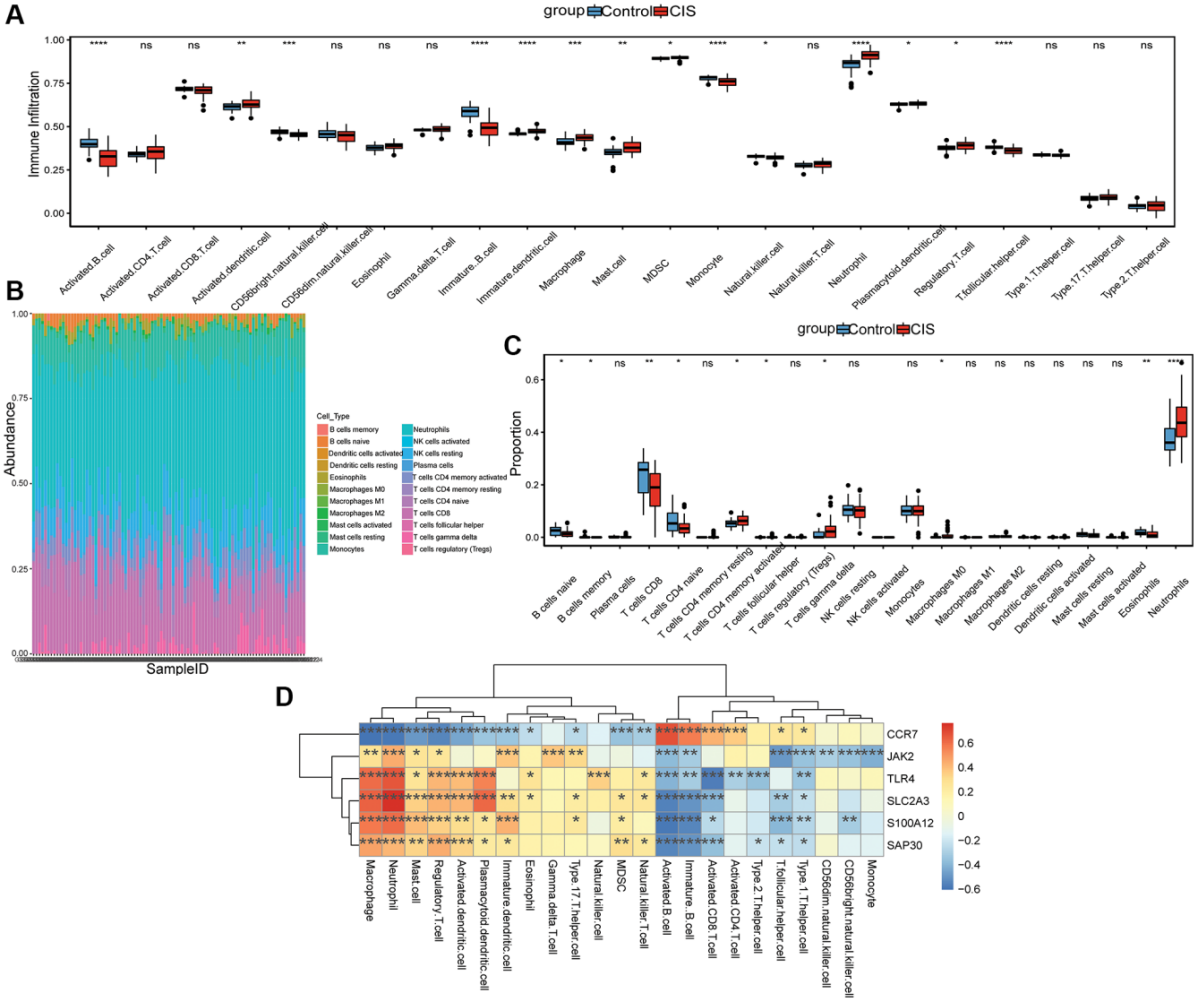
**Analysis of immune cell infiltration.** (**A**) The level of immune cell infiltration was analyzed by ssGSEA method; (**B**) Stacked histogram of the proportion of each immune cell in the sample analyzed by CIBERSORT method; (**C**) Box diagram of the proportion of each immune cell in the sample analyzed by CIBERSORT method; (**D**) Correlation between hub mRNAs and immune cell infiltration. Red and blue represent positive and negative correlations, respectively. ^*^P < 0.05, ^**^P < 0.01, ^***^P < 0.001, ^****^P < 0.0001, Abbreviation: ns: no significant significance.

The ssGSEA analysis showed that the infiltration levels of Activated dendritic cell, Immature dendritic cell, Macrophage, Mast cell, MDSC, Neutrophil, Plasmacytoid dendritic cell and Regulatory T cell in CIS group were higher than that in control group, while the infiltration level of Activated B cell, CD56 bright natural killer cell, Immature B cell, Monocyte, Natural killer cell and T follicular helper cell in CIS group was lower (Figure 6A). In addition, we also evaluated the proportion of immune cells according to CIBERSORT method, and the proportion of Neutrophils in the CIS group was also higher (Figure 6B and 6C). Pearson correlation analysis found that Macrophage, Activated B cell, Neutrophil, Immature B cell, Regulatory T cell, Mast cell and Type 1 T helper cell were all correlated with the 6 hub mRNAs (Figure 6D). Macrophage has the highest negative correlation with CCR7 (−0.61), while Neutrophil has the highest positive correlation with SLC2A3 (0.76).

### Construction of TFs regulatory network and ceRNA regulatory network based on hub mRNAs

**Figure 7:**
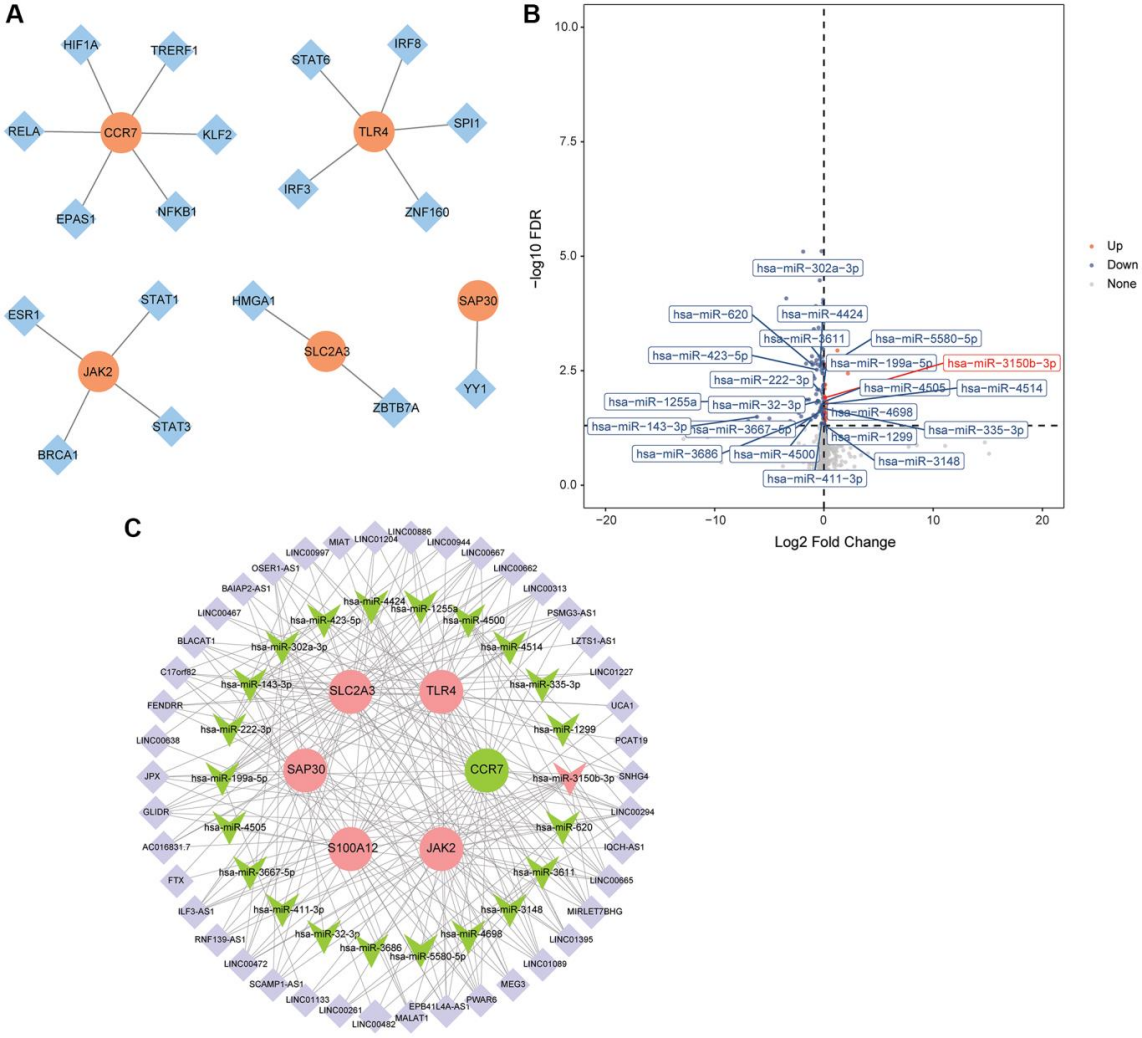
**Construction of TFs regulatory network and ceRNA regulatory network.** (**A**) TFs regulatory network; (**B**) Volcano map of DEmiRNAs in the GSE95204 dataset; (**C**) CeRNA regulatory network.

TFs related to hub mRNAs were queried based on TRRUST database, and the results were imported into Cytoscape to construct a regulatory network. The results showed that S100A12 had no related TF, CCR7 had 6 related TFs (HIF1A, TRERF1, KLF2, NFKB1, EPAS1 and RELA), TLR4 had 5 related TFs (IRF3, IRF8, SPI1, STAT6 and ZNF160), JAK2 had 4 related TFs (BRCA1, ESR1, STAT1 and STAT3), SLC2A3 had 2 related TFs (HMGA1 and ZBTB7A), and SAP30 had 1 related TF (YY1) (Figure 7A). A total of 623 targeted miRNAs were predicted for 6 hub mRNAs based on ENCORI and miRDB databases. 127 DEmiRNAs (33 up-regulated and 94 down-regulated) were obtained by differential expression analysis of miRNAs in the GSE95204 dataset (Figure 7B). Then, the intersection of DEmiRNAs negatively regulated with hub mRNAs and targeted miRNAs were screened out. 22 intersection miRNAs were obtained. The lncRNAs associated with intersection miRNAs were searched based on ENCORI database, and then the correlation between these lncRNAs and hub mRNAs was predicted. Subsequently, 42 lncRNAs associated with hub mRNAs were screened out. The correlation between CCR7 and EPB41L4A-AS1 was the highest (0.80), followed by SLC2A3 and LINC01089 (−0.67). Finally, the ceRNA regulatory network was constructed based on 42 lncRNAs, 22 intersection miRNAs and 6 hub mRNAs (Figure 7C).

### Drug prediction and molecular docking

**Figure 8:**
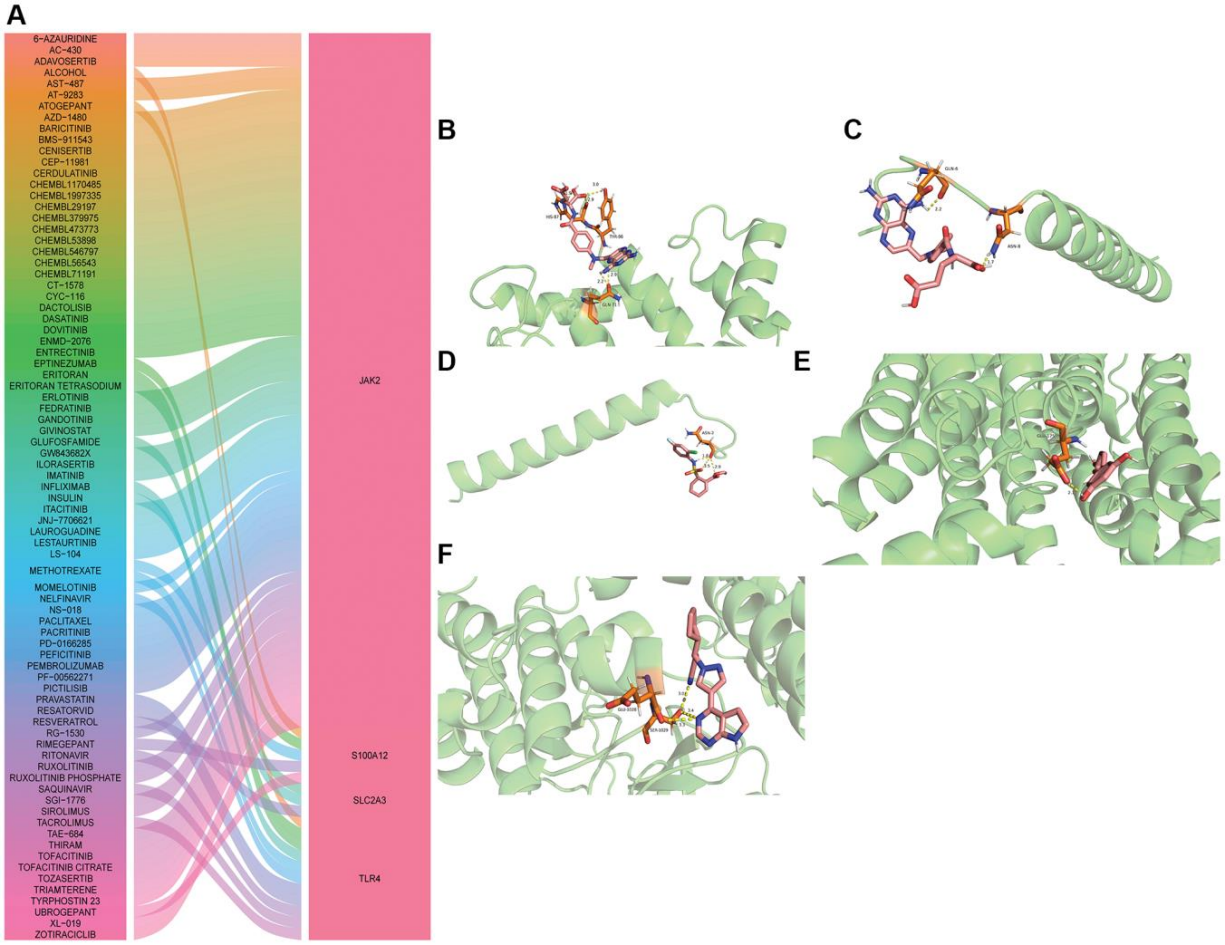
**Drug prediction and molecular docking of hub mRNAs.** (**A**) Drug prediction of hub mRNAs; (**B**) Molecular docking of methotrexate and S100A12; (**C**) Molecular docking of methotrexate and TLR4; (**D**) Molecular docking of resatorvid and TLR4; (**E**) Molecular docking of resveratrol and SLC2A3; (**F**) Molecular docking of ruxolitinib and JAK2.

Based on DGIdb database, related drugs of hub mRNAs were predicted, but only related drugs of JAK2, S100A12, SLC2A3 and TLR4 were obtained (Figure 8A). The search of these drugs found that ruxolitinib, methotrexate, resveratrol and resatorvid play an important role in the treatment of brain injury. Subsequently, the molecular docking of ruxolitinib, methotrexate, resveratrol and resatorvid and their action hub mRNAs was performed. When the binding energy between the drug and the target protein is the lowest, it shows the best conformation and the interaction mode between the drug molecule and the target protein (Figure 8B–8F and Table 1). The lowest binding energies between drugs and target proteins were all less than −1.19423 kcal/mol, which implies that ruxolitinib, methotrexate, resveratrol and resatorvid may play a role in the treatment of diseases by acting on target genes.

**Table 1:** Minimum binding energy in molecular docking between hub mRNAs and drug molecule.

**Medicine**	**Gene**	**Binding Energy**
METHOTREXATE	S100A12	−1.92
METHOTREXATE	TLR4	−1.38
RESATORVID	TLR4	−2.47
RESVERATROL	SLC2A3	−3.83
RUXOLITINIB	JAK2	−3.3

### *In vitro* cell validation

**Figure 9:**
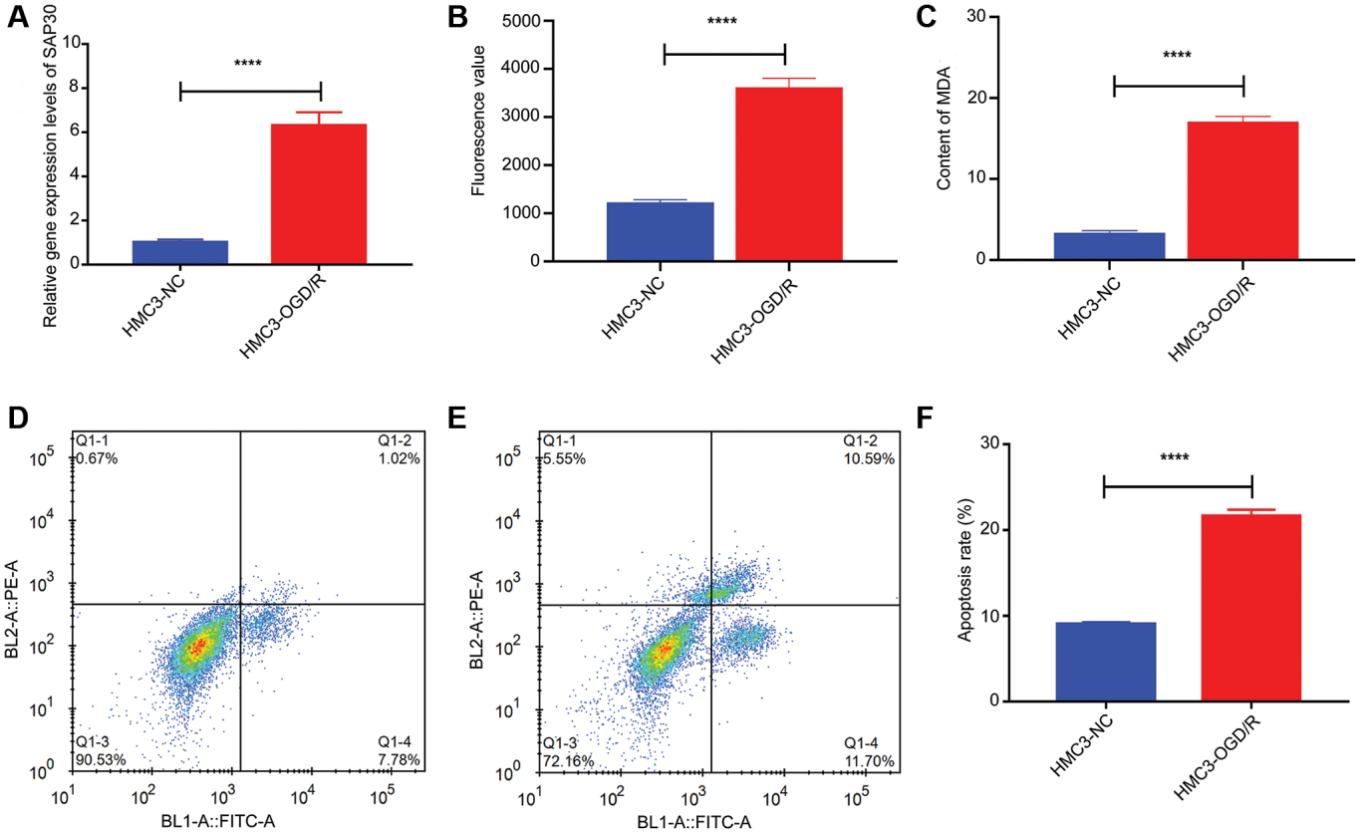
**The relative content of SAP30, ROS and MDA and the apoptosis rate in HMC3-OGD/R model group and HMC3 control group.** (**A**) The relative expression level of SAP30 in HMC3-OGD/R model group and HMC3 control group was detected by real time-PCR; (**B**) Fluorescence value of ROS in HMC3-OGD/R model group and HMC3 control group; (**C**) Content of MDA in HMC3-OGD/R model group and HMC3 control group; (**D**) Apoptosis rate in HMC3 control group was detected by flow cytometry; (**E**) Apoptosis rate in HMC3-OGD/R model group was detected by flow cytometry; (**F**) Histogram of apoptosis rate in HMC3-OGD/R model group and HMC3 control group. ^****^P < 0.0001; Abbreviations: HMC3-NC: HMC3 control group; HMC3-OGD/R HMC3-OGD/R model group. Q1-2 and Q1-4 quadrants represent late apoptotic cells and early apoptotic cells, respectively.

**Figure 10:**
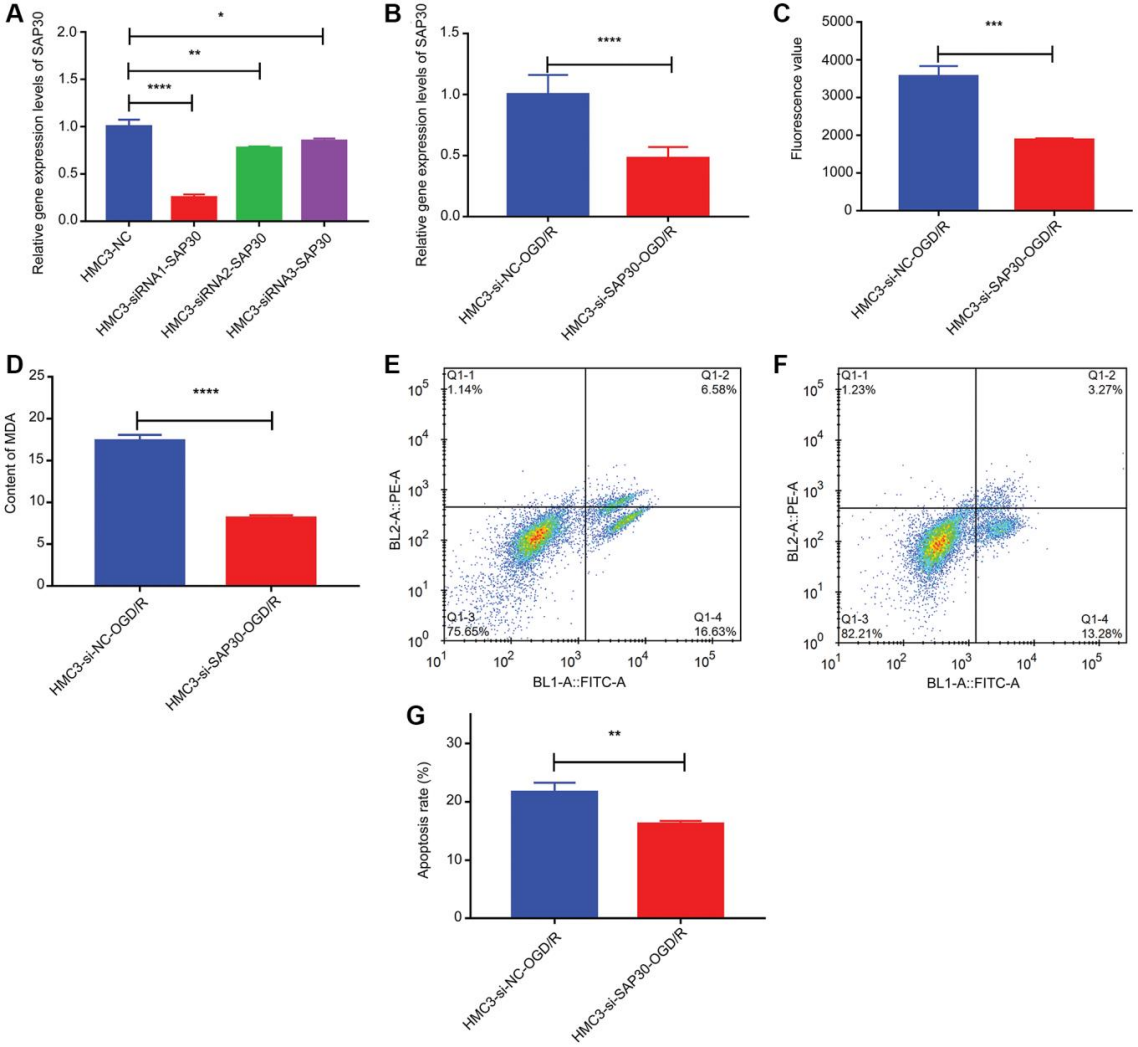
**The relative content of SAP30, ROS and MDA and the apoptosis rate in HMC3-si-NC-OGD/R group and HMC3-si-SAP30-OGD/R group.** (**A**) Real time-PCR was used to detect the expression of SAP30 to screen out effective interference targets in HMC3 cell; (**B**) The relative expression level of SAP30 in HMC3-si-NC-OGD/R group and HMC3-si-SAP30-OGD/R group; (**C**) Fluorescence value of ROS in HMC3-si-NC-OGD/R group and HMC3-si-SAP30-OGD/R group; (**D**) Content of MDA in HMC3-si-NC-OGD/R group and HMC3-si-SAP30-OGD/R group; (**E**) Apoptosis rate in HMC3-si-NC-OGD/R group was detected by flow cytometry; (**F**) Apoptosis rate in HMC3-si-SAP30-OGD/R group was detected by flow cytometry; (**G**) Histogram of apoptosis rate in HMC3-si-NC-OGD/R group and HMC3-si-SAP30-OGD/R group. ^*^P < 0.05, ^**^P < 0.01, ^***^P <0.001, ^****^P < 0.0001. Q1-2 and Q1-4 quadrants represent late apoptotic cells and early apoptotic cells, respectively.

CCR7, S100A12, SAP30 and SLC2A3 were selected for real time-PCR validation to detect the relative expression levels in HMC3-OGD/R model group and HMC3 control group. The results showed that only SAP30 was significantly increased in the HMC3-OGD/R model group and the expression trend was consistent with the results of bioinformatics analysis (Figure 9A). Therefore, SAP30 was selected for the follow-up experiment. In addition, the relative expressions of ROS and MDA and apoptosis of cells in HMC3-OGD/R model group and HMC3 control group were also detected. The results showed that the contents of ROS and MDA in the HMC3-OGD/R model group were higher than those in the HMC3 control group (Figure 9B and 9C), and the HMC3-OGD/R model group also had higher apoptosis rate (Figure 9D–9F). SAP30 was selected to knockdown in HMC3 cells. Then, the expression of SAP30 was detected by real time-PCR to screen out effective interference targets. The results showed that target 1 had the best knockdown effect (Figure 10A), so siRNA1-SAP30 was selected for subsequent experiments. Subsequently, the relative expressions of SAP30, ROS and MDA and apoptosis of cells in HMC3-si-NC-OGD/R group and HMC3-si-SAP30-OGD/R group were also detected. The results showed that the expression levels of SAP30, ROS and MDA in HMC3-si-SAP30-OGD/R group were significantly decreased (Figure 10B–10D), and the apoptosis rate was also significantly decreased (Figure 10E–10G). These results suggest that inhibition of SAP30 expression in CIS may reduce cell apoptosis and inhibit ROS and MDA production, thus playing a regulatory role in disease progression.

## DISCUSSION

In this study, differential expression analysis found that GSE58294 and GSE16561 datasets had 6,145 and 648 DEmRNAs, respectively. Based on the GSE58294 dataset, a total of 6 modules were identified by WGCNA, among which the red module had the highest negative correlation with CIS and the green module had the highest positive correlation with CIS. Subsequently, 1,127 candidate hub mRNAs were identified in red module and green module according to GS >0.2 and MM >0.5. The intersection of the DEmRNAs in the GSE58294 dataset, the DEmRNAs in the GSE16561 dataset, the candidate hub mRNAs in the WGCNA and the set of IRGs and HRGs were taken. A total of 26 intersection mRNAs were obtained. KEGG analysis showed that intersection mRNAs were significantly enriched in cytokine-cytokine receptor interaction, hepatitis B and JAK-STAT signaling pathway. A study found that CCL2 is highly expressed in ischemic stroke tissues, which may promote the progression of ischemic stroke by activating chemokine signaling pathway and cytokine-cytokine receptor interaction pathway [21]. Comprehensive analysis of m6A methylation in human ischemic stroke blood showed that cytokine-cytokine receptor interaction is also a significantly enriched signaling pathway [22]. A study of Taiwan nationals found that hepatitis B virus is associated with a reduced risk of acute ischemic stroke [23]. IL-21R plays a key role in neuronal protection through the JAK-STAT signaling pathway in ischemic stroke [24]. JAK2-STAT3 signaling pathway plays a protective role in improving inflammation, oxidative stress and neuronal apoptosis after cerebral ischemia-reperfusion injury mediated by interleukin-22 [25]. At present, the specific molecular mechanism of cytokine-cytokine receptor interaction, hepatitis B and JAK-STAT signaling pathway in CIS are still unclear, and a large number of experiments are needed for further study.

6 hub mRNAs (CCR7, JAK2, S100A12, SAP30, SLC2A3 and TLR4) were identified from 26 intersection mRNAs based on machine learning. A study showed that CCR7 mRNA expression was reduced in patients with traumatic brain injury within 24 hours of injury [26]. The expression of CCR7 was also down-regulated in peripheral blood during the acute phase of ischemic stroke [27]. As a key factor of JAK2/STAT3 signal pathway, JAK2 is involved in regulating neuroinflammation of cerebral ischemic injury, and can also mediate the polarization of microglia [28–30]. Inhibition of TLR4 may play a role in reducing inflammation in ischemic stroke [31]. Loss of TLR4 increases the level of alternative Neutrophils and is associated with neuroprotection after stroke [32]. KEGG analysis showed that CCR7 was enriched in the cytokine-cytokine receptor interaction, JAK2 was enriched in the hepatitis B and JAK-STAT signaling pathway, and TLR4 was enriched in the hepatitis B. Therefore, we speculated that CCR7, JAK2 and TLR4 may play a role in CIS by regulating related pathways. High plasma S100A12 levels on admission are associated with a poor functional outcome in patients with acute ischemic stroke [33]. SLC2A3 also known as GLUT3, is significantly up-regulated in the penumbra after cerebral ischemia [34]. In addition, GLUT3 may mediate nerve protection [35]. So far, no relevant studies on SAP30 in brain injury have been found.

This study may be the first to discover abnormal expression of SAP30 in CIS. SAP30 was a HRG screened from the MSigDB database. As the most vulnerable organ in the human body, the brain needs an adequate and timely supply of oxygen and energy. Previous studies have shown that the mortality and recurrence rates of ischemic stroke increase with the duration of hypoxia [36, 37]. Therefore, exploring the molecular mechanism of hypoxia in CIS can help in disease management. The outcome of ischemic stroke largely depends on the amount of hypoxia-related neuronal death in the affected brain area [38]. Microglial cell is the first response cells of ischemic brain injury. In ischemic cerebrovascular disease, microglial cell is the key to neuronal damage and remodeling [39]. HMC3 cells, a type of microglial cell in the brain. So far, HMC3 cell is also commonly used in research on cerebrovascular diseases [40–42]. In this study, HMC3 cells were also selected for *in vitro* experiments to investigate the potential role of SAP30. The results showed that knockdown of SAP30 could significantly reduce the contents of ROS and MDA in HMC3-OGD/R model, and inhibit apoptosis. These results suggest that inhibition of SAP30 expression in CIS may reduce cell apoptosis and inhibit ROS and MDA production, thus playing a regulatory role in disease progression.

Subsequently, SVM, RF and DT classification models were constructed based on 6 hub mRNAs. The results showed that SVM, RF and DT classification models all had high AUC values, which indicated that these three classification models had high diagnostic accuracy. Moreover, the AUC of 6 hub mRNAs was less than SVM, RF and DT classification models, which implied that the diagnostic accuracy of classification models were higher than that of single hub mRNAs. To further understand the molecular mechanisms of the hub mRNAs that constitute classification models, we constructed TFs and ceRNA regulatory networks. In the TFs regulating network, we found that HIF-1α is related to CCR-7. The HIF-1α is considered to be a key regulator of oxygen homeostasis. It is also involved in mediating neuroprotective effects in ischemic stroke [43]. Moreover, CCR7 is abnormally expressed in ischemic stroke [27]. Therefore, we speculated that HIF-1α may be involved in mediating the influence of CCR7 on CIS progression. It also suggests that there may be some potential links between hypoxia and immune regulation, but the specific mechanism needs further study. In the ceRNA regulating network, we found that CCR7 and EPB41L4A-AS1 have the highest correlation (0.80), followed by SLC2A3 and LINC01089 (−0.67). One study found that EPB41L4A-AS1 is closely related to type 2 diabetic mellitus, and EPB41L4A-AS1 knockdown can enhance the inflammatory response [44]. Zheng et al. identified 11 hub lncRNAs in ischemic stroke based on subpathway-LNCE method, including EPB41L4A-AS1 [45]. Furthermore, LINC01089 has been found to be a potential therapeutic target for acute ischemic stroke [46]. In this study, we also found that EPB41L4A-AS1 and LINC01089 were correlated with hsa-miR-3611 and hsa-miR-4424, respectively. Therefore, we hypothesized that the hsa-miR-3611/EPB41L4A-AS1/CCR7 axis and hsa-miR-4424/LINC01089/SLC2A3 axis play a moderating role in the progression of CIS. So far, no relevant research on hsa-miR-3611 and hsa-miR-4424 has been found in brain diseases. The specific mechanism of hsa-miR-3611/EPB41L4A-AS1/CCR7 axis and hsa-miR-4424/LINC01089/SLC2A3 in CIS needs a lot of experiments for further verification. In addition, the correlation between hub mRNAs and immune cells was also analyzed. The results showed that Macrophage has the highest negative correlation with CCR7 (−0.61), while Neutrophil has the highest positive correlation with SLC2A3 (0.76). Macrophages are one of the major cellular contributors to neuroinflammation [47]. The blocking of Macrophage infiltration may also be associated with reduced the infarct size and mitigated neurological deficits in mice after ischemic stroke [48]. Stroke attracts Neutrophils to injured brain tissue, where they can disrupt the integrity of the blood-brain barrier and exacerbate lesions [49]. Neutrophils are a precursor of brain injury after ischemic stroke and are associated with poor prognosis after stroke [50]. Therefore, we speculated that CCR7 and SLC2A2 may also be involved in regulating the immunomodulatory effects of Macrophages and Neutrophils and play a role in the brain injury of CIS.

In this study, we also obtained drugs related to JAK2, S100A12, SLC2A3 and TLR4 based on the DGIdb database. Ruxolitinib, formerly known as INCB018424 or INC424, is a potent inhibitor of JAK1 and JAK2 [51]. A previous study has shown that ruxolitinib treatment can improve the neurological score, reduce the infarct size, improve cerebral edema and inhibit the expression of pro-inflammatory factors in stroke [28]. At a certain dose, short-term methotrexate may reduce the risk of ischemic stroke in rheumatoid arthritis patients [52]. Resveratrol preconditioning significantly improved neurological function, reduced infarct volume and reduced neuronal apoptosis *in vivo* and *in vitro* after stroke [53, 54]. In the rat model of ischemic stroke, resveratrol treatment not only significantly reduced infarction, but also improved motor and cognitive function. In addition, resveratrol pretreatment also improved the markers of oxidative stress [55]. Resatorvid, also known as TAK-242, is a small molecule specific inhibitor of TLR4 signaling that inhibits the production of inflammatory mediators by binding to TLR4 [56]. TAK-242 is able to cross the blood-brain barrier and block TLR4 signaling, mediating the expression of inflammatory cytokines, and protecting the brain from I/R-induced acute damage [57]. When the binding energy between the drug and the target protein is the lowest, it shows the best conformation and the interaction mode between the drug molecule and the target protein. In this study, the lowest binding energies between drugs and target proteins were all less than −1.19423 kcal/mol. Binding energy less than −1.19423 kcal/mol is the basis for screening candidate targets of active ingredients [18, 19]. This again proves that ruxolitinib, methotrexate, resveratrol and resatorvid have important value in the treatment of CIS. In addition, other drugs identified may also play a role in CIS treatment, but the specific mechanism of action still needs further study.

However, this experiment still has a certain degree of limitations. First, the identified hub mRNAs and classification diagnostic models lack clinical verification. Therefore, a large number of clinical samples need to be collected in the later period for further research. Secondly, the specific mechanism of key signaling pathways and important molecules in CIS is still unclear, so a large number of experimental studies are needed in the later stage.

## CONCLUSION

In conclusion, the identification of hub miRNAs and the construction of classification models provide a theoretical basis for the diagnosis and management of CIS. Moreover, inhibition of SAP30 expression in CIS may reduce cell apoptosis and inhibit ROS and MDA production, thus playing a regulatory role in disease progression.

## Supplementary Materials

Supplementary Figures

Supplementary Tables
