# Supplementary material for: Reveal the correlation between hub hypoxia/immune-related genes and immunity and diagnosis, and the effect of SAP30 on cell apoptosis, ROS and MDA production in cerebral ischemic stroke
Source: Aging (Albany NY). 2023 Dec 27;15(24):15161–82. doi: 10.18632/aging.205339 (PMC10781503; doi:10.18632/aging.205339)
Supplement: Supplementary Figures [file aging-15-205339-s001.pdf]

SUPPLEMENTARY FIGURES

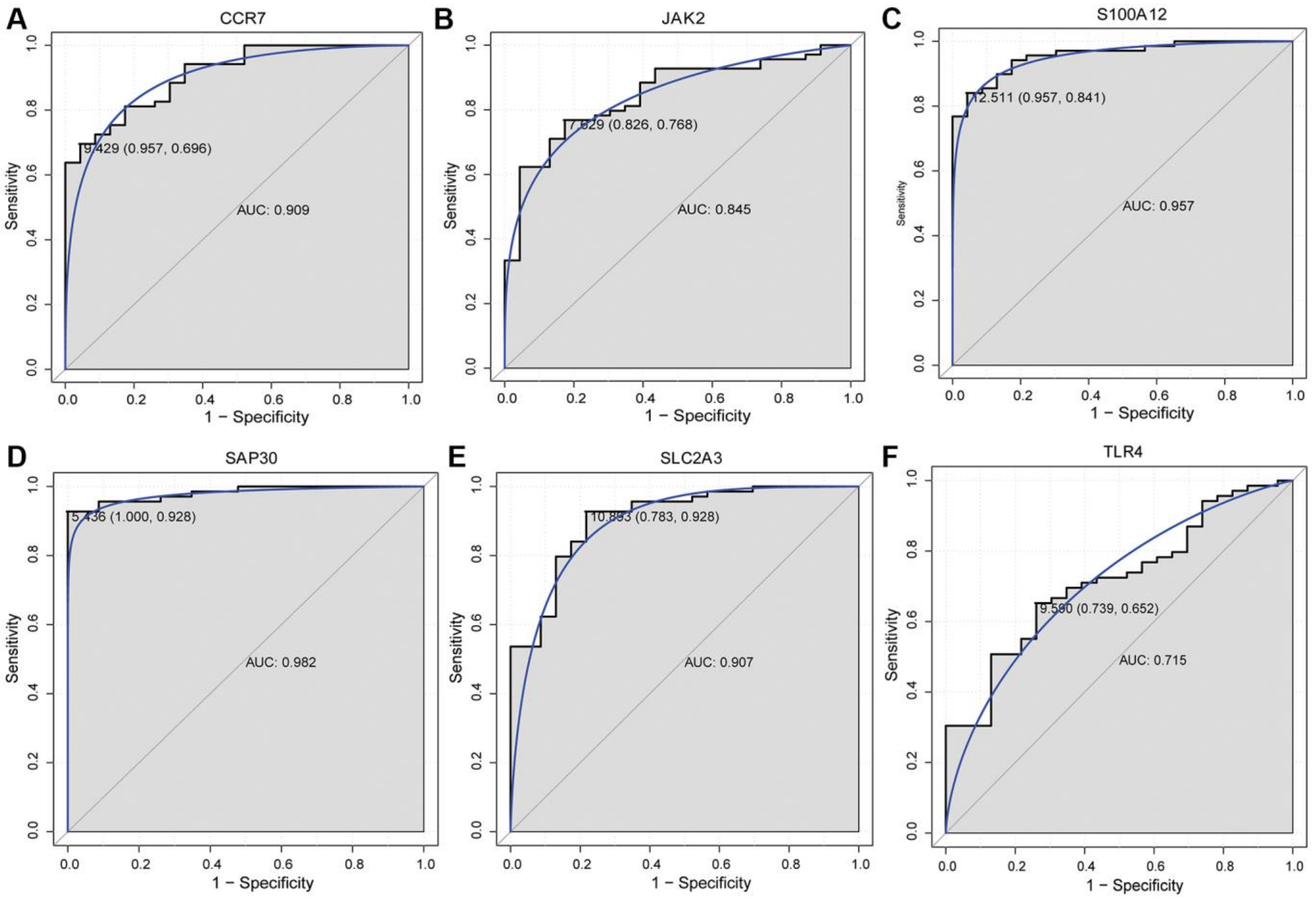

Supplementary Figure 1. The ROC curves of CCR7 (**A**), JAK2 (**B**), S100A12 (**C**), SAP30 (**D**), SLC2A3 (**E**) and TLR4 (**F**) in the GSE58294 dataset.

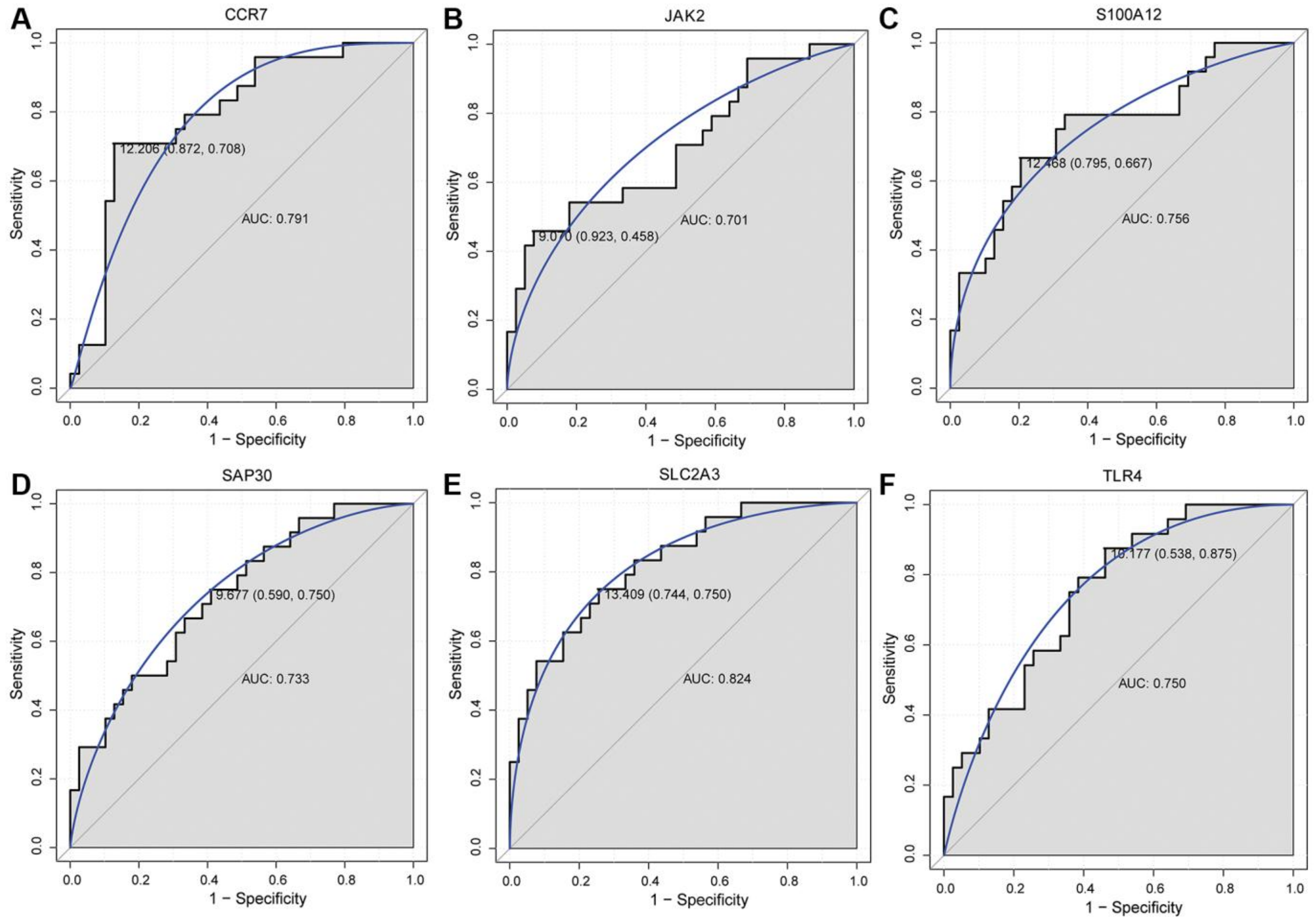

Supplementary Figure 2. The ROC curves of CCR7 (**A**), JAK2 (**B**), S100A12 (**C**), SAP30 (**D**), SLC2A3 (**E**) and TLR4 (**F**) in the GSE16561 dataset.

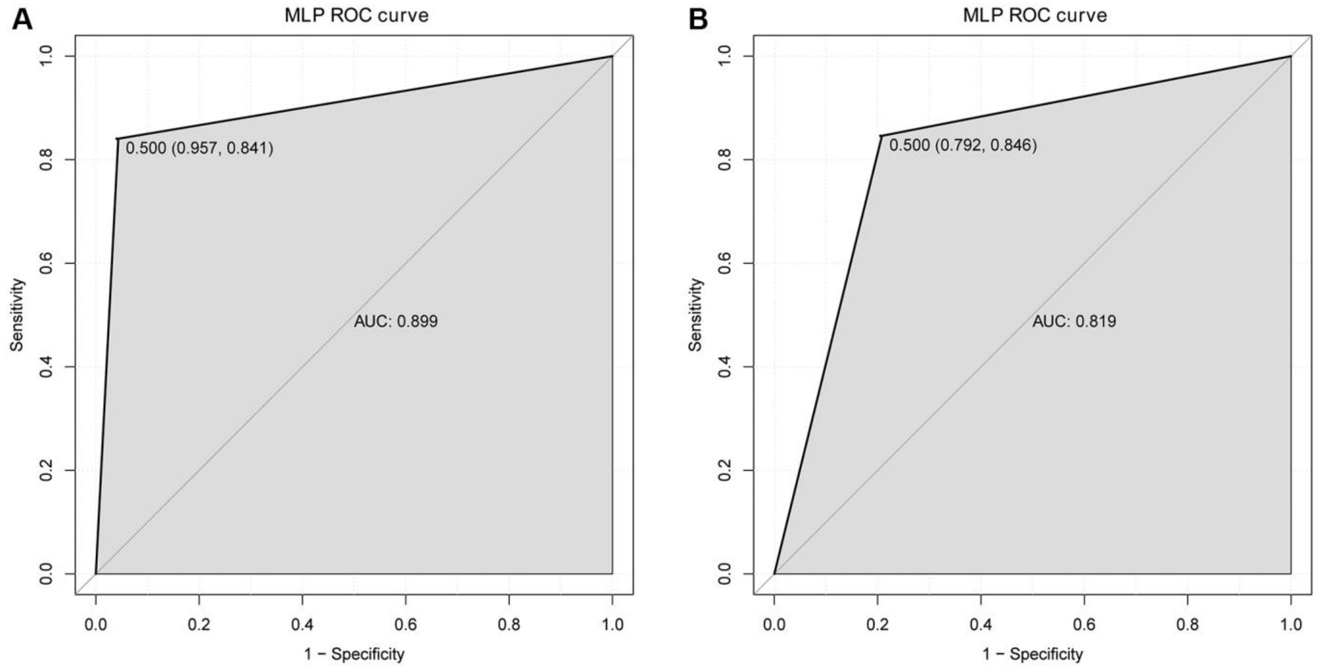

Supplementary Figure 3. The ROC curves of MLP classification model in GSE58294 (**A**) and GSE16561 (**B**) datasets.
