# Supplementary material for: Reveal the correlation between hub hypoxia/immune-related genes and immunity and diagnosis, and the effect of SAP30 on cell apoptosis, ROS and MDA production in cerebral ischemic stroke
Source: Aging (Albany NY). 2023 Dec 27;15(24):15161–82. doi: 10.18632/aging.205339 (PMC10781503; doi:10.18632/aging.205339)
Supplement: Supplementary Tables [file aging-15-205339-s002.pdf]

SUPPLEMENTARY TABLES

Supplementary Table 1. Detailed information of the datasets selected in this study.

GEO ID	Platform	Year	Omics	Sample type	Author	Samples size
GSE58294	GPL570 [HG-U133_Plus_2] Affymetrix Human Genome U133 Plus 2.0 Array	2014	mRNA	blood	StamovaBB	CIS: Control = 69:23
GSE16561	GPL6883 Illumina HumanRef-8 v3.0 expression beadchip	2010	mRNA	blood	BarrTL	CIS: Control = 39:24
GSE95204	GPL18058 Exiqon miRCURY LNA microRNA array, 7th generation [miRBase v18, condensed Probe_ID version]	2017	miRNA	blood	Deng Z	CIS: Control = 3:3

Supplementary Table 2. Primer sequences used for the RT-PCR.

Primer name	Primer sequence (5′ to 3′)
Human ACTB/Actin-F (internal reference)	5′-GACAGGATGCAGAAGGAGATTACT-3′
Human ACTB/Actin-R (internal reference)	5′-TGATCCACATCTGCTGGAAGGT-3′
CCR7-F	5′-GTGATCGGCTTTCTGGTCCC-3′
CCR7-R	5′-GACCACAGCGATGATCACCTTG-3′
S100A12-F	5′-CTTCCACCAATACTCAGTTCG-3′
S100A12-R	5′-GCAAGCTCCTTTGTAAGCA-3′
SAP30-F	5′-AACGCCAGCTTCAGCAAGA-3′
SAP30-R	5′-TGTAAAGATGCCTTGCGCTCT-3′
SLC2A3-F	5′-GCTCTTTCCAATTTGGCTACAAC-3′
SLC2A3-R	5′-GCATTTCCCTTGTCCGTCA-3′
